# Insulin-induced serine 22 phosphorylation of retinoid X receptor alpha is dispensable for adipogenesis in brown adipocytes

**DOI:** 10.1080/21623945.2020.1747352

**Published:** 2020-04-05

**Authors:** Jacob Ardenkjær-Larsen, Kaja Rupar, Goda Sinkevičiūtė, Patricia S. S. Petersen, Julia Villarroel, Morten Lundh, Romain Barrès, Atefeh Rabiee, Brice Emanuelli

**Affiliations:** Novo Nordisk Foundation Center for Basic Metabolic Research, Faculty of Health and Medical Sciences, University of Copenhagen, Copenhagen, Denmark

**Keywords:** Retinoid X receptor alpha, insulin, phosphorylation, adipose tissue, transcriptional regulation

## Abstract

Insulin action initiates a series of phosphorylation events regulating cellular differentiation, growth and metabolism. We have previously discovered, in a mass spectrometry-based phosphoproteomic study, that insulin/IGF-1 signalling induces phosphorylation of retinoid x receptor alpha (RXRα) at S22 in mouse brown pre-adipocytes. Here, we show that insulin induces the phosphorylation of RXRα at S22 in both brown precursor and mature adipocytes through a pathway involving ERK, downstream of IRS-1 and −2. We also found that RXRα S22 phosphorylation is promoted by insulin and upon re-feeding in brown adipose tissue *in vivo*, and that insulin-stimulated S22 phosphorylation of RXRα is dampened by diet-induced obesity. We used *Rxra* knockout cells re-expressing wild type (WT) or S22A non-phosphorylatable forms of RXRα to further characterize the role of S22 in brown adipocytes. Knockout of *Rxra* in brown pre-adipocytes resulted in decreased lipid accumulation and adipogenic gene expression during differentiation, and re-expression of *Rxra*WT alleviated these effects. However, we observed no significant difference in cells re-expressing the *Rxra*S22A mutant as compared with the cells re-expressing *Rxra*WT. Furthermore, comparison of gene expression during adipogenesis in the WT and S22A re-expressing cells by RNA sequencing revealed similar transcriptomic profiles. Thus, our data propose a dispensable role for RXRα S22 phosphorylation in adipogenesis and transcription in differentiating brown pre-adipocytes.

## Introduction

Insulin action in adipose tissue is one of the most potent signals regulating cell metabolism and differentiation [1]. Insulin resistance, defined at the molecular level by defective signalling in response to insulin, plays an important role in metabolic disorders such as obesity and type II diabetes [2]. The elucidation of the molecular mechanisms mediating insulin signalling is therefore essential for the development of therapeutic strategies to treat metabolic disorders.

Activation of insulin and insulin-like growth factor 1 (IGF-1) receptors (IR/IGF-1 R) by their ligands initiates a cascade of phosphorylation events controlling cellular differentiation, growth and metabolism [3]. To obtain a better understanding of the molecular mechanisms mediating insulin/IGF-1 action, we previously performed a mass spectrometry-based phosphoproteomic study using brown pre-adipocytes. In this study, we found retinoid x receptor alpha (RXRα) to be phosphorylated on serine 22 (S22) with a 2.9-fold induction upon IGF-1 stimulation [4]. The RXRα S22 phosphorylation site is located in an autonomous, ligand-independent transcriptional activation domain in the N-terminal A/B region of RXRα [5]. This domain is called ‘activation function 1’ (AF-1) and is required for the transduction of retinoic acid signals during development [6]. It has been reported that RXRα constitutive (without ligand) phosphorylation on S22 is required for the anti-proliferative effect of retinoic acid and induction of several retinoic acid-responsive genes [7]. Phosphorylation at this site is the most commonly detected post-translational modification of RXRα in high-throughput studies in mouse, human and rat [8]. Among these studies, one study found increased RXRα S22 phosphorylation in human neuroblastoma, which had significantly elevated insulin receptor signalling[9], and another study detected RXRα S22 phosphorylation in response to insulin in mature 3T3-L1 adipocytes [10]. However, the physiological relevance and impact of this phosphorylation event in adipocytes have not been investigated.

RXRα is a ligand-activated transcription factor that belongs to the superfamily of nuclear hormone receptors. It is expressed in many tissues, such as adipose tissue, liver, kidney, spleen and muscle [11], and plays a role in development as well as in regulating the physiological functions of differentiated adult tissues [12]. Several nuclear receptors heterodimerize with RXRα which gives it a unique modulatory role across multiple signalling pathways [13]. Genome-wide profiling of peroxisome proliferator-activated receptor-gamma (PPARγ) and RXRα binding in 3T3-L1 adipocytes has demonstrated that the PPARγ:RXRα heterodimer binds to hundreds of genomic locations in differentiating and mature adipocytes [14], suggesting a role for this heterodimer in adipogenesis and adipocyte metabolism. Consistently, selective ablation of RXRα in adipose tissue results in impaired lipolysis during fasting, impaired adipogenesis and resistance to obesity [15].

Here, we show that the phosphorylation of RXRα at S22 is promoted by insulin in brown precursor and mature adipocytes, and in brown adipose tissue (BAT) *in vivo* following insulin injection or re-feeding. Interestingly, insulin-induced RXRα S22 phosphorylation is dampened by diet-induced obesity. However, our *in vitro* studies indicate that RXRα S22 phosphorylation is dispensable for adipogenesis and does not influence gene expression throughout differentiation.

## Results

### Insulin triggers phosphorylation of RXRα at S22 in brown pre-adipocytes and brown adipose tissue

**Figure 1.**
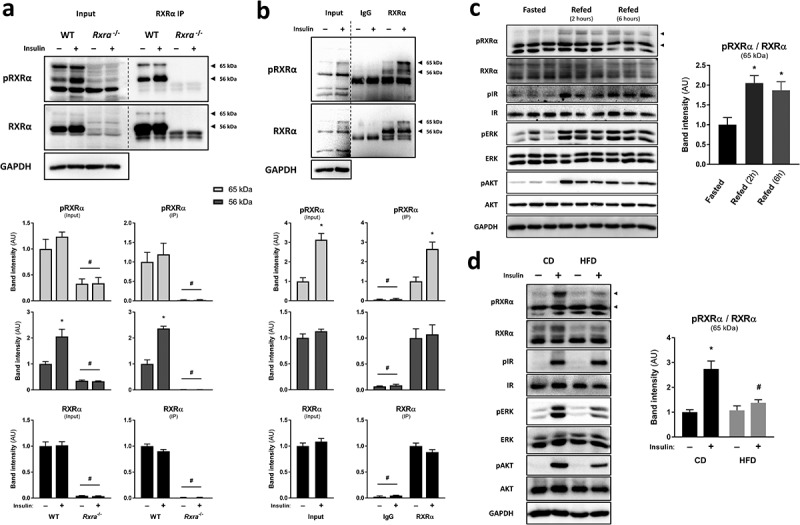
Insulin stimulates phosphorylation of RXRα at S22 *in vitro* and *in vivo*. Representative immunoblots with quantified pRXRα (56 kDa and 65 kDa, separately) and RXRα (56 kDa and 65 kDa together) band intensities in brown pre-adipocytes and mouse BAT presented as mean ± SE. Immunoprecipitation (IP) was performed using control IgG or RXRα antibody (D6H10). A different RXRα antibody (D-20) was used for immunoblotting. Lysate input or immunoprecipitated RXRα were prepared from (a) WT or *Rxra*^−/-^ brown pre-adipocytes treated with insulin or vehicle; *n* = 3; or (b) BAT from mice injected with insulin or saline; *n* = 5; two-tailed t-test or two-way ANOVA with Šídák’s multiple comparison tests. (c) BAT from mice that were fasted or refed for 2 or 6 h; *n* = 6; one-way ANOVA and Dunnett’s multiple comparison test. (d) BAT from mice that were fed with CD or HFD and were injected with insulin or saline; *n* = 5–6; two-way ANOVA and Šídák’s multiple comparison test. All phosphoprotein band intensities are normalized to the total amount of each respective protein, except in immunoprecipitation experiments where pRXRα and RXRα are quantified separately. Asterisk (*) represents a significant difference (*p* < 0.05) from vehicle-treated control cells, fasted mice or saline-injected control mice. Hash (#) represents a significant difference (*p* < 0.05) from WT cells, BAT RXRα IP or insulin-injected CD mice

To test whether RXRα phosphorylation on S22, detected in our phosphoproteomic study, occurs in response to insulin, we applied an antibody directed against phosphorylated-S22 RXRα (pRXRα) for protein analysis using protein lysates from brown pre-adipocytes stimulated with insulin or vehicle. Three main bands in the molecular weight range of RXRα were detected in wild type (WT) cells using the phosphospecific antibody, with the middle one corresponding to the expected molecular weight of RXRα (Figure 1(a)). Notably, the intensity of the two bands of higher molecular weight was dramatically reduced when immunoblotting with either pRXRα or total RXRα antibody in *Rxra*^−/-^ cells (Figure 1(*a*)), or following siRNA-mediated knockdown of *Rxra* in brown pre-adipocytes (Supplementary Figure 1(a)). Furthermore, the same two bands were detected following RXRα immunoprecipitation in WT cells, but not in *Rxra*^−/-^ cells, by immunoblotting with either pRXRα or total RXRα antibody (Figure 1(a)). Additionally, we confirmed the specificity of the phosphospecific antibody towards the phosphorylated form of the RXRα S22 motif using a peptide competition assay with the phosphorylated peptide used to generate the pRXRα antibody and its non-phosphorylated analog (Supplementary Figure 1(b)). Collectively, these data indicate that two bands correspond to RXRα, and, importantly, that RXRα S22 phosphorylation is increased upon insulin stimulation (Figure 1(a); Supplementary Figure 1(a, b)).

Next, we sought to determine if RXRα is phosphorylated on S22 *in vivo*. BAT was taken from mice after injection with saline or insulin, and protein lysates were analysed by immunoblotting following immunoprecipitation. Interestingly, we observed increased RXRα phosphorylation on S22 in response to insulin, here at the level of the higher molecular weight (Figure 1(b)). Similarly, we observed increased S22 phosphorylation of RXRα in BAT upon 2 or 6 h of refeeding, highlighting that RXRα is phosphorylated on S22 under normal physiological conditions (Figure 1(c)). Increased phosphorylation of IR, AKT and ERK in response to feeding suggests a response to endogenous insulin (Supplementary Figure 1(c)). Furthermore, to assess the effect of obesity-associated insulin resistance on RXRα S22 phosphorylation in BAT, we measured RXRα S22 phosphorylation in BAT from either lean mice fed with control diet (CD) or diet-induced obese and hyperglycaemic mice fed a high-fat diet (HFD) for 8 weeks (Supplementary Figure 1(d)). In BAT, RXRα S22 phosphorylation in response to insulin was blunted in HFD-fed animals, as compared to CD-fed mice (Figure 1(d)). This was paralleled with lower AKT and ERK activation in the HFD-fed mice, indicative of insulin resistance in this tissue (Supplementary Figure 1(e)). Thus, RXRα S22 phosphorylation occurs in response to insulin and refeeding in BAT, and is compromised in conditions of insulin resistance.

### Insulin triggers S22 phosphorylation of RXRα via ERK activation in precursor and mature adipocytes

**Figure 2.**
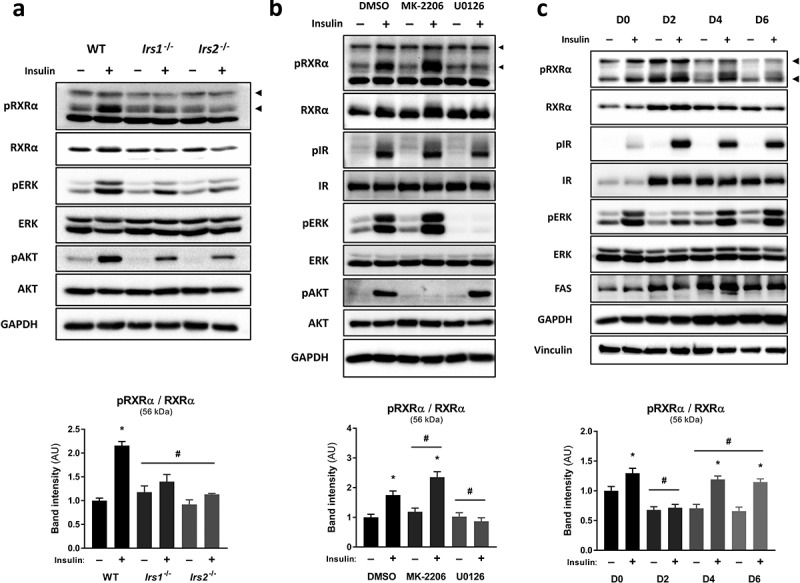
Insulin stimulates S22 phosphorylation of RXRα via ERK in precursor and mature adipocytes. Representative immunoblots with quantified pRXRα band intensities presented as mean ± SE; *n* = 3; two-way ANOVA with Šídák’s and Dunnett’s multiple comparison tests. (a) WT, *Irs1*^−/-^ or *Irs2*^−/-^ brown pre-adipocytes were treated with insulin or vehicle. (b) Brown pre-adipocytes were pre-treated with vehicle (DMSO), AKT inhibitor (MK-2206) or MEK inhibitor (U0126), and then stimulated with insulin or vehicle. (c) Brown pre-adipocytes at day 0, 2, 4 or 6 of differentiation were stimulated with insulin or vehicle. All phosphoprotein band intensities are normalized to the total amount of each respective protein. Asterisk (*) represents a significant difference (*p* < 0.05) from vehicle-treated control cells. Hash (#) represents a significant difference (*p* < 0.05) from WT cells, DMSO-treated cells or D0 cells

In order to investigate the signalling events mediating RXRα phosphorylation at S22 in response to insulin, we assessed RXRα S22 phosphorylation in brown pre-adipocytes depleted of IRS-1 and −2, the main substrates downstream of IR/IGF-1 R. We found that insulin- and IGF-1-induced RXRα S22 phosphorylation was abolished in *Irs1*^−/-^ and *Irs2*^−/-^ cells, as compared to WT brown pre-adipocytes, and this was accompanied by reduced phosphorylation of AKT and ERK (Figure 2(a); Supplementary Figure 2(a, b)). These results indicate that IRS-1 and IRS-2 are both required for maximal RXRα S22 phosphorylation, and suggest that AKT or ERK mediate this effect.

To investigate the potential contribution of these two major insulin-activated pathways in mediating phosphorylation of RXRα at S22, pharmacological inhibition was used to suppress the kinase activities of AKT and ERK using MK-2206 and U0126, respectively, without affecting IR activity (Supplementary Figure 2(c)). ERK inhibition in brown pre-adipocytes prevented insulin-mediated RXRα S22 phosphorylation (Figure 2(b)). In contrast, there was enhanced insulin-mediated ERK and RXRα S22 phosphorylation upon AKT inhibition (Figure 2(b); Supplementary Figure 2(c)). Thus, ERK is the main kinase involved in mediating insulin-induced RXRα S22 phosphorylation in brown pre-adipocytes.

To study how phosphorylation of RXRα on S22 is regulated throughout adipogenesis, brown pre-adipocytes were induced to differentiate and were stimulated with insulin or vehicle across differentiation at four time points (day 0, 2, 4 or 6). Increased maturation of the brown pre-adipocytes was indicated by increases in IR, FAS and GAPDH abundance (Figure 2(c)) [16]. RXRα S22 phosphorylation occurred in response to insulin at all time points except for day 2 (Figure 2(c)), and this was paralleled by a similar phosphorylation pattern of ERK (Supplementary Figure 2(d)). These results indicate that insulin mediates S22 phosphorylation of RXRα in both brown precursor and mature adipocytes, and suggests that RXRα S22 phosphorylation may play an important role during adipogenesis, and to modulate adipocyte functions.

### RXRα S22 phosphorylation is dispensable for adipogenesis

**Figure 3.**
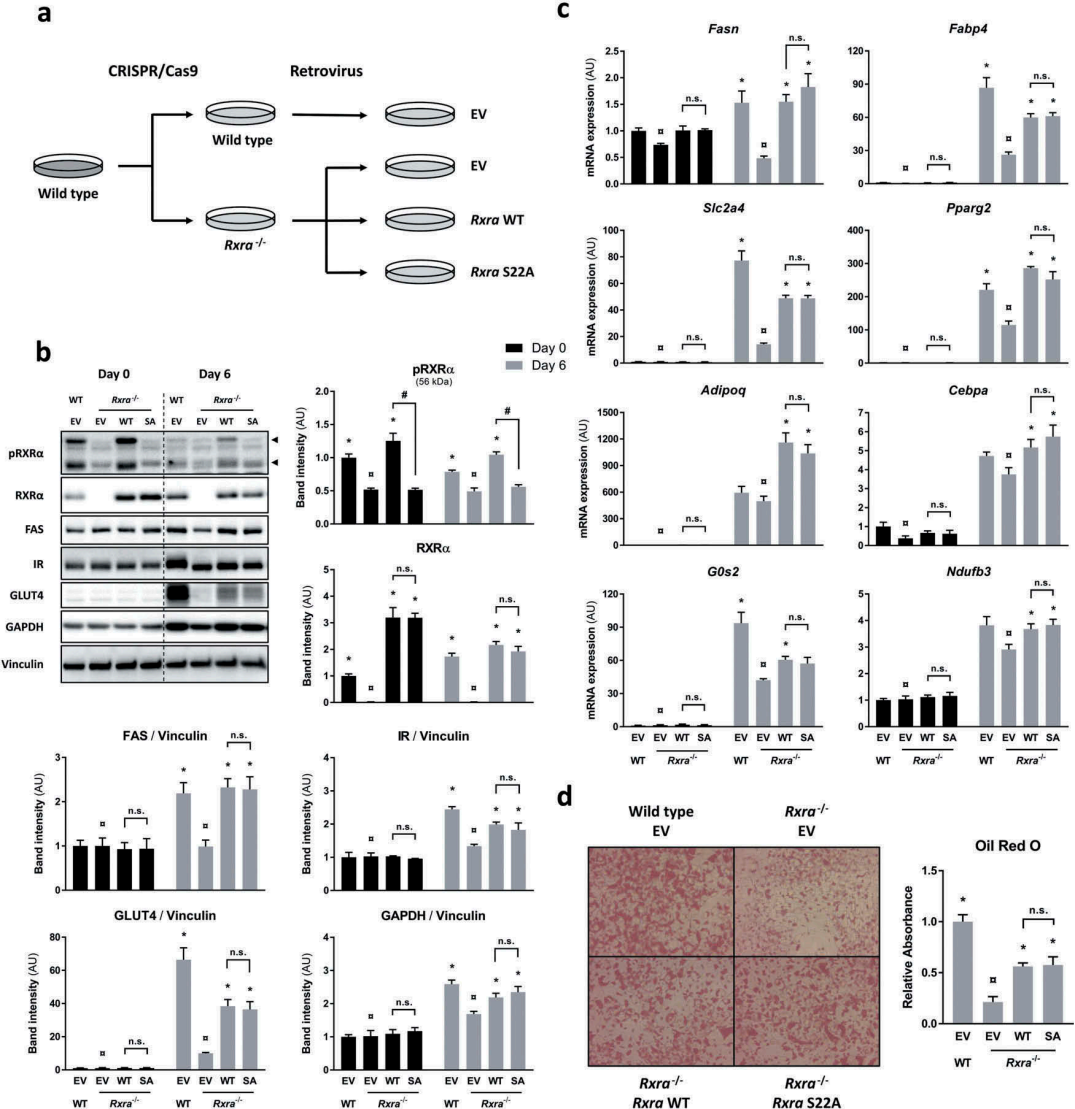
Adipocyte maturation occurs independently of RXRα phosphorylation at S22. (a) WT brown pre-adipocytes were transfected with EV, and *Rxra*^−/-^ cells were transfected with EV, *Rxra*WT or *Rxra*SA. (b) Immunoblots with quantified band intensities at day 0 or day 6 of differentiation; *n* = 4; two-way ANOVA and Tukey’s multiple comparison test. All protein band intensities are normalized to vinculin, except for pRXRα and RXRα, which are quantified separately. (c) Gene expression analysis by RT-qPCR showing mRNA levels of adipogenic markers at day 0 or day 6 of differentiation; *n* = 4; two-way ANOVA and Tukey’s multiple comparison test. (d) Lipid staining with Oil Red O at day 6 of differentiation; *n* = 3; one-way ANOVA and Tukey’s multiple comparison test. The presented values are mean ± SE. Asterisk (*) represents a significant difference (*p* < 0.05) from sample indicated with the scarab (¤) sign. Other comparisons are indicated with a hash (#) representing statistical significance (*p* < 0.05) and n.s. as an abbreviation for non-significance (*p* > 0.05)

To assess the role of RXRα S22 phosphorylation in mediating insulin action, we generated cells in which phosphorylation of RXRα on S22 was prevented. This was accomplished by reconstituting *Rxra*^−/-^ brown pre-adipocytes with WT or S22A mouse *Rxra* (Figure 3(a)). While insulin induced S22 phosphorylation of RXRα in two separate *Rxra*^−/-^ clones re-expressing *Rxra*WT (Supplementary Figure 3(a)), there was no detectable S22 phosphorylation of RXRα in *Rxra*^−/-^ cells re-expressing *Rxra*SA in response to insulin (Supplementary Figure 3(b)) or adipogenic induction (Figure 3(b); Supplementary Figure 3(c)), despite comparable abundance of RXRα protein. Given the adipogenic action of insulin and the role of RXRα in adipogenesis through dimerization with the master regulator PPARγ, we next compared the ability of these brown pre-adipocyte cell lines to differentiate. The adipogenic potential of each cell line was assessed by comparing expression of different markers of differentiation, such as protein levels of FAS, IR, GLUT4 and GAPDH (Figure 3(b)) and expression of *Fasn, Fabp4, Slc2a4, Pparg2, Adipoq, Cebpa, G0s2* and *Ndufb3* mRNA (Figure 3(c)). As expected, these classic adipogenic markers were more abundant (*p* < 0.05; overall effect of days using two-way ANOVA) in fully differentiated adipocytes (day 6) than at the pre-adipocyte stage (day 0). At day 6, there was a lower expression of adipogenic markers in the *Rxra*^−/-^ cells compared with WT controls, indicative of a defect in adipogenesis. Upon re-introduction of *Rxra*WT in *Rxra*^−/-^ cells, the expression of these markers increased to reach levels close to those of WT cells, indicating that exogenously delivered RXRα was able to restore the adipogenic capacity of *Rxra*^−/-^ cells. Remarkably, re-expression of *Rxra*SA resulted in restored expression of adipogenic genes to a similar level as in cells re-expressing *Rxra*WT. Consistent with this, lipid staining of differentiated brown adipocytes at day 6 was lower in *Rxra*^−/-^ cells and partially rescued in cells re-expressing *Rxra*WT, with equal lipid accumulation in *Rxra*WT and *Rxra*SA (Figure 3(d)). Collectively, these data indicate that RXRα WT and SA are equally capable of rescuing adipogenesis in *Rxra*^−/-^ cells.

### RXRα S22 phosphorylation does not affect gene expression during adipogenesis

**Figure 4.**
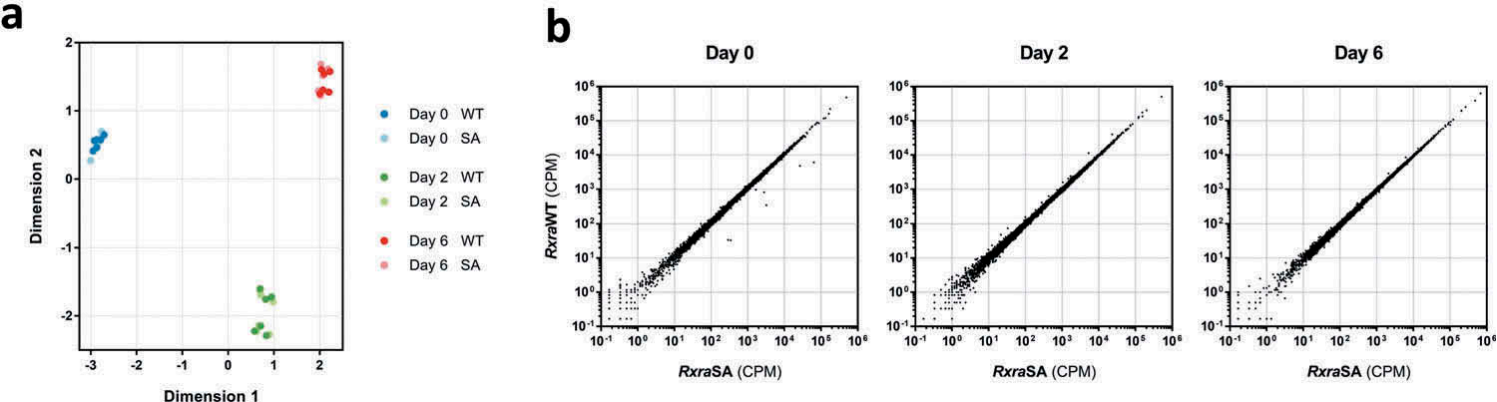
RXRα phosphorylation at S22 is not involved in the regulation of gene expression during brown adipocyte differentiation. Two different *Rxra*^−/-^ brown pre-adipocyte clones re-expressing *Rxra*WT or *Rxra*SA were differentiated to day 0, 2 or 6, followed by high-throughput RNA sequencing; *n* = 3 for each cell line. (a) Similarities in gene expression profiles between the four different lines, and across differentiation, are visualized using MDS. (b) Scatter plots illustrating transcript counts per million (CPM) at each time point

We then hypothesized that RXRα S22 phosphorylation may play a role in the transcriptional control of differentiating adipocytes. Two different *Rxra*^−/-^ clones, each re-expressing *Rxra*WT or *Rxra*SA, were induced to differentiate, and high-throughput RNA sequencing was performed to compare the transcriptomic profiles at 0, 2 or 6 days following induction of differentiation. Multidimensional scaling (MDS) revealed a clear separation between the three time points (Figure 4(a)). Strikingly, the transcriptomes of the cells re-expressing either *Rxra*WT or *Rxra*SA were indistinguishable at each day of the differentiation process. Furthermore, there was no significant statistical difference in RNA levels of any genes between the cells re-expressing *Rxra*WT or *Rxra*SA, with transcript counts approximately equal for every gene in the two cell populations at all three time points (Figure 4(b)). Performing the data analysis for each *Rxra*^−/-^ clone separately did not further reveal any significant difference at any time point. There was little overlap between the two *Rxra*^−/-^ clones for the genes approaching significance for differential expression, illustrating that they are unlikely to be caused by S22A mutation (Supplementary Figure 4(a, b)). Collectively, our data indicate that RXRα S22 phosphorylation does not impact transcriptional regulation in brown adipocytes during adipogenesis *in vitro*.

## Discussion

Brown adipocytes are important for the regulation of metabolic homoeostasis, and further insight into the signalling events controlling thermogenic adipocyte differentiation and function is essential for understanding and treating metabolic disorders. During adipogenesis, external adipogenic signals activate a cascade of transcription factors that are critical for differentiation. These transcription factors include the two nuclear hormone receptors PPARγ and RXRα that heterodimerize and regulate transcription of various genes involved in adipogenesis, as well as lipid and glucose metabolism [17]. Our study sought to uncover a role of insulin-stimulated RXRα phosphorylation at S22 in brown adipocytes.

Using a newly generated antibody specifically raised against the phosphorylated form of RXRα, we show that RXRα phosphorylation on S22 occurs in brown precursor and mature adipocytes in response to insulin or IGF-1. This is consistent with phosphoproteomic studies that detected the S22 phosphorylation in WT-1 brown pre-adipocytes in response to IGF-1 [4], and in mature 3T3-L1 adipocytes in response to insulin [10]. In the present study, we additionally show that S22 phosphorylation of RXRα occurs in BAT, indicating its physiological relevance. Moreover, our data indicate that this phosphorylation is dysregulated with insulin resistance, revealing a potential role in pathophysiological situations. Interestingly, insulin-induced RXRα S22 phosphorylation *in vivo* was detected by immunoblotting as a band of higher molecular weight than in cultured cells. It was previously reported that hyperphosphorylation of RXRα at four residues (distinct from S22) causes an upward shift in electrophoretic mobility [18], which is characteristic of proline-directed sites such as these four phosphorylation sites. This suggests that insulin induces RXRα phosphorylation on S22 in a non-hyperphosphorylated state *in vitro*, while S22 phosphorylation *in vivo* occurs in conjunction with hyperphosphorylation. Our pharmacological inhibition results indicate that ERK is necessary for insulin-induced phosphorylation of RXRα at S22. As this site matches the consensus sequence for proline-directed kinases such as mitogen-activated protein kinases (MAPKs), this further suggests that ERK itself may catalyse the S22 phosphorylation reaction. Interestingly, a missense single nucleotide polymorphism (SNP) in the *RXRA* gene (rs55836231) [19] is predicted to cause a loss-of-phosphorylation at S21 (the corresponding phosphorylation site in humans) [20] by disrupting the phosphorylation motif.

As both insulin action and ERK activation promote adipogenesis [21], insulin-stimulated S22 phosphorylation of RXRα by ERK is suggestive of a role for RXRα S22 phosphorylation in adipocyte differentiation. Furthermore, RXRα is the heterodimerization partner of PPARγ in adipose cells, constituting a master transcriptional complex coordinating adipocyte differentiation. Confirming a role for RXRα in brown adipocyte differentiation, our newly generated *Rxra*^−/-^ cells exhibit impaired differentiation compared with WT cells. This is consistent with a previous study reporting that adipocyte-specific knockout of *Rxra* results in poor adipocyte differentiation [15]. Re-introduction of WT RXRα into *Rxra*^−/-^ cells largely restored adipogenesis. However, re-expressing *Rxra* with an S22A mutation resulted in similar lipid content and gene expression pattern as when re-expressing *Rxra*WT. This indicates that RXRα S22 phosphorylation is dispensable for the differentiation of brown pre-adipocytes into mature adipocytes and does not influence gene expression during adipogenesis. PPARγ itself is phosphorylated by MAPKs in the AF-1 transactivation domain in 3T3-L1 cells which reduces ligand-dependent transcriptional activity [22] and inhibits adipogenic activity [23]. Conversely, our data indicate that RXRα S22 phosphorylation in the AF-1 domain does not affect RXRα:PPARγ heterodimer function in adipocyte transcriptional control. In *Ras*-transformed keratinocytes, phosphorylation of human RXRα at S260 affects its cellular localization and its binding to chromatin and to the vitamin D receptor (VDR) [24]. Like S22, phosphorylation at this site occurs via MAPK [25]. This raises the possibility that S22 phosphorylation may play a role in modulating RXRα function by altering, e.g, localization or partnering of RXRα, or its transcriptional activity. Phosphorylation of RXRα at S22 has previously been shown to affect the expression of retinoic acid-responsive genes in F9 embryocarcinoma cells [7]. This role for RXRα S22 phosphorylation in transcriptional regulation was observed in response to retinoic acid, suggesting that RXRα S22 phosphorylation modulates RXRα activity only in the presence of its ligand. Alternatively, because RXRα is phosphorylated on multiple sites, it is possible that a potential outcome of S22 phosphorylation in regulating RXRα function might require phosphorylation on other residues. Our results revealed that insulin-mediated phosphorylation of S22 in BAT is associated with hyperphosphorylation, which could indicate that S22 phosphorylation operates differently *in vivo*, where it may potentially interact with other phosphorylation events. In addition, the dampening of insulin-induced S22 phosphorylation observed in BAT from HFD-fed mice could indicate a potential role in the pathogenesis of insulin resistance. Similarly, dysregulation of gene expression upon phosphorylation of PPARγ on S273 by cyclin-dependent kinase 5 (CDK5) is particularly apparent in the context of obesity [26].

In conclusion, our findings reveal that RXRα is phosphorylated on S22 in BAT and in cultured brown adipocytes in response to insulin. We show that S22 phosphorylation is dispensable for the regulation of transcription during adipogenesis in brown adipocytes. Yet, whether RXRα S22 phosphorylation affects RXRα function and the regulation of metabolic processes *in vivo* remains to be determined, in particular in obese conditions.

## Materials and methods

### Animal housing and diet

All mice were housed at 22°C under daily 12 h light/dark cycles. Ten 9–11-week-old male C57BL/6 J mice were fasted for 4 h and anaesthetized, followed by retro-orbital injection of 1 unit insulin/saline for 5 min (5 mice per group). For the fasting/refeeding challenge, 9-week old male mice were fasted overnight, refed for either 2 or 6 h (6 mice per group), and euthanized [27]. For the high-fat diet challenge, 6-week old male mice were fed with either control diet (D12450B, Research Diets, Inc., USA) or high-fat diet (D12492, Research Diets, Inc., USA) for 8 weeks. The mice were fasted for 2 h, prior to injection with insulin/saline (5–6 mice per group), and euthanized. Tissues were collected right after euthanasia and immediately snap frozen in liquid nitrogen. All animal experiments were approved by the Danish authorities (permit number 2015-15-0201-00728) at the University of Copenhagen.

### Cell culture

Wild-type (WT), *Irs1*^−/-^ and *Irs2*^−/-^ brown preadipocyte cell lines were a kind gift from C. Ronald Kahn [28]. Cells were cultured in Dulbecco’s modified Eagle’s medium (DMEM) containing 10% foetal bovine serum and 1% penicillin-streptomycin (PS), in humidified incubators at 37°C and 5% CO_2_. For differentiation, pre-adipocytes were grown to confluence (day 0) in culture medium supplemented with 20 nM insulin and 1 nM triiodothyronine (differentiation medium). Confluent cells were incubated for 2 d in differentiation medium further supplemented with 1 μM dexamethasone, 0.5 mM isobutylmethylxanthine and 0.125 mM indomethacin. Subsequently, the cells were maintained in differentiation medium. Culture medium was changed every 2 d, and cell culturing experiments were performed in independent replicates.

For stimulation experiments, cells were starved for 4 h in DMEM with 1% PS and 0.1% bovine serum albumin. Cells were stimulated with 100 nM insulin or IGF-1, or vehicle for 5 min before lysis. For kinase inhibition, cells were pre-treated with 10 μM U0126-EtOH (Selleck Chemicals), 10 μM MK-2206 (Active Biochem) or vehicle (0.1% DMSO) for 1 h before stimulation.

### Transient knockdown

Knockdown of *Rxra* was performed by RNA interference in brown pre-adipocytes. At 80% confluency, the cells were transfected with a pool of four siRNAs targeting *Rxra* (LQ-050277-01-0002, Dharmacon) or a pool of four non-targeting control (NTC) siRNAs (D-001810-10-05, Dharmacon) using Lipofectamine RNAiMAX Transfection Reagent (Thermo Fisher) and Opti-MEM Reduced Serum Medium (Thermo Fisher) according to the manufacturer’s instructions. Cells were incubated for 2 d before lysis and subsequent protein analysis.

### Targeted genome editing

The CRISPR/Cas9 system was used for knocking out the *Rxra* gene in a clonal cell line derived from WT pre-adipocytes. Single guide RNAs (sgRNAs) were designed using the CRISPR Design Tool (https://zlab.bio/guide-design-resources) and delivered via a sgRNA-expressing plasmid. The targeting sequence (5ʹ-GCCATGGAGCCTCGACCCGT-3ʹ) of the sgRNA was ligated into a pSpCas9(BB)-2A-GFP (PX458) vector [29], which was replicated using chemically competent *E. coli*. Correct insertion was verified by Sanger sequencing. The Nucleofector 2b Device (Lonza) was used to deliver the plasmids into the WT cell clone by electroporation using Cell Line Nucleofector Kit R (Lonza Group). After 2 d, the cells were separated based on green fluorescent protein (GFP) intensity using fluorescence-activated cell sorting. Monoclonal cell populations were obtained from the GFP-positive cells by isolation of single cells via dilution, and frameshift mutations were confirmed by Sanger sequencing. Two independent *Rxra*^−/-^ clones were subsequently used for experiments.

### Subcloning and mutagenesis

Mouse *Rxra* cDNA, reverse transcribed from brown pre-adipocyte mRNA, was cloned into overhanging 3ʹ deoxythymidine residues of pCR2.1-TOPO (Invitrogen) after PCR amplification with specific primers carrying appropriate restriction sites at their 5′ ends. The full-length cDNA was validated by Sanger sequencing and subcloned into EcoRI and SalI sites of pBabe Hygro (#1765, Addgene) [30], a retroviral expression plasmid, generating pBabe Hygro Mouse Rxra. A missense mutation was introduced in the plasmid using the Q5 Site-Directed Mutagenesis Kit (New England Biolabs) to generate a plasmid coding for RXRα with a serine-to-alanine substitution at residue 22.

### Retroviral transduction

Homozygous *Rxra* knockout cells were transduced with empty vector (EV) or retroviral vector containing WT or S22A mutant *Rxra*. A CRISPR/Cas9-targeted cell clone without *Rxra* frameshift indels was transduced with EV and was used as control.

HEK293 FT cells were transiently transfected with pBabe Hygro Mouse Rxra, pUMVC (#8449, Addgene) [31] and pCAG-Eco (#35617, Addgene) in the ratio of 8:8:1 using the TransIT-X2 Dynamic Delivery System. After incubation for 3 d, virus-containing medium was transferred to brown pre-adipocytes together with 8 μg/mL polybrene. Antibiotic selection with hygromycin (200 μg/mL) started 3 d after virus infection and until all uninfected control cells were eliminated.

### Protein immunoblotting

Cells were washed with cold phosphate-buffered saline (PBS) and lysed in RIPA buffer containing protease and phosphatase inhibitors. Lysate was collected and centrifuged for 10 min at 4°C and 16,100 rcf to pellet insoluble material. The pellet was discarded, and protein concentrations of cell lysates were determined with bicinchoninic acid assay. The samples were diluted to equal protein concentrations and mixed with Laemmli sample buffer. Proteins were separated by SDS-PAGE and transferred onto a PVDF membrane. Immunostaining was performed using the primary antibodies shown in Supplementary Table 1 and HRP-conjugated secondary antibodies (Bio-Rad). A rabbit polyclonal antibody specific to phosphoserine 22 in RXRα was produced by Thermo Fisher Scientific with a synthetic phosphopeptide corresponding to residues 18–28 of mouse RXRα ([C]-SSLNS(p)PTGRGS-amide).

In the peptide competition assay, the phospho-RXRα (pRXRα) antibody was pre-incubated for 1 h at room temperature with either water, the immunizing phosphopeptide that the antibody was raised against, or the non-phosphorylated analog peptide. The samples were electrotransferred together in three copies which were cut apart and immunostained in parallel with the antibody solutions. The membrane pieces were assembled for chemiluminescent detection.

### Immunoprecipitation

Brown pre-adipocytes were lysed according to the immunoblotting procedure, and samples were diluted to equal protein concentrations. Samples were then incubated with RXRα (D6H10) antibody (#3085, Cell Signalling Technology) or normal rabbit IgG (#2729, Cell Signalling Technology) overnight at 4°C while rotating. Immunocomplexes were captured by incubation with Protein A Sepharose beads for another 4 h. The beads were washed 3 times in TBST and eluted using Laemmli buffer. For tissue samples, the beads were washed in lysis buffer instead. Immunoblotting was performed using a secondary antibody (ab99697, Abcam) specific to the light chain of rabbit IgG to avoid co-detection of IgG heavy chain from the primary antibody.

### Gene expression analysis

RNA from cultured cells was extracted using RNeasy Kit (Qiagen), and cDNA was synthesized using iScript cDNA Synthesis Kit (Bio-Rad) according to the manufacturer’s instructions. Real-time quantitative PCR was performed using Brilliant III Ultra-Fast SYBR Green QPCR Master Mix (Agilent) and the CFX384 Real-Time PCR Detection System (Bio-Rad) according to the supplier’s manual. Each cDNA sample was run in technical triplicates, and the 2^−ΔΔCt^ method was used for relative quantification with normalization to the sum of *Rn18 s* and *Tbp*. Primer sequences are shown in Supplementary Table 2.

### Lipid staining

Differentiated cells were washed twice in PBS followed by fixation in 10% formaldehyde for 30 min. Fixed cells were washed twice in distilled water and then equilibrated with 60% isopropanol in water for 5 min. Lipids were stained by incubating cells for 20 min with a freshly prepared solution of 0.3% Oil Red O and 60% isopropanol in water. Finally, the cells were washed in distilled water to remove excess stain and were covered with water when viewed under the microscope.

For relative quantification, the cells were washed twice for 5 min in 60% isopropanol while agitating. Oil Red O stain was extracted with 100% isopropanol for 20 min while agitating. Absorbance was read at 518 nm, and the absorbance of 100% isopropanol was subtracted (background).

### RNA sequencing

Cell lysis and phase separation were performed using TRIzol Reagent (Invitrogen) according to the manufacturer’s instructions. The upper part of the aqueous phase was transferred to a new tube and mixed with an equal volume of 70% RNase-free ethanol. The RNA was purified using RNeasy Kit (Qiagen), including the DNase digestion step, according to the manufacturer’s instructions. RNA quantity and quality were assessed using NanoDrop 2000 (Thermo Scientific) and RNA 6000 Nano Kit (Agilent), respectively. Library preparation was performed using TruSeq Stranded Total RNA Library Prep Gold (Illumina) with RNAClean XP Kit (Agencourt) for RNA purification, AMPure XP Kit (Agencourt) for DNA purification and SuperScript III Reverse Transcriptase (Invitrogen) for cDNA synthesis. DNA quantity and quality were assessed using Qubit dsDNA HS Assay Kit (Invitrogen) and High Sensitivity DNA Kit (Agilent), respectively. High-throughput sequencing was performed using the NextSeq 500 Sequencing System (Illumina) and the NextSeq 500/550 High Output Kit v2 (75 cycles).

### Transcriptomic analysis

Paired-end reads were checked for quality with FastQC and aligned to the CRCm38.p6 GENCODE primary assembly, version M21 using STAR v2.7.0d [32]. Read summation onto genes of the comprehensive gene annotation on the aforementioned primary assembly was performed by featureCounts v1.6.2 [33]. Differential gene expression was calculated by edgeR v3.24.3 [34] using the model ~0+group + block, where group was a factor with information about genotype (WT or S22A) and time (d 0, 2 or 6), and block encoded the two independent *Rxra*^−/-^ cell lines. Differential expression was performed using edgeR quasi-likelihood test on different contrasts. Contrasts were contemplating differences between genotypes either at different times, e.g. S22A_D0 – WT_D0, or at different ranges of time, that is the interaction between genotype and time, e.g. (SA_D2 – SA_D0) – (WT_D2 – WT_D0). RNA-seq data was deposited in GEO (GEO: GSE147687).

### Statistical analysis

Statistical analyses were performed using GraphPad Prism 7. Statistical significance was tested using two-tailed t-test or analysis of variance (ANOVA). Šídák’s, Dunnett’s or Tukey’s multiple comparison tests were applied as follow-up tests for two-way ANOVA, as recommended by Prism 7. Dunnett’s comparison test was applied after one-way ANOVA. Data were considered significant if *p* ≤ 0.05 and are presented as mean ± standard error (SE) of the mean.

## Supplementary Material

Supplemental Material
