# Supplementary material for: Insulin-induced serine 22 phosphorylation of retinoid X receptor alpha is dispensable for adipogenesis in brown adipocytes
Source: Adipocyte. 2020 Apr 5;9(1):142–52. doi: 10.1080/21623945.2020.1747352 (PMC7153655; doi:10.1080/21623945.2020.1747352)
Supplement: Supplemental Material [file KADI_A_1747352_SM0410.zip › Supplementary Figures and Tables_clean.docx]

**Supplementary Figure 1**

**Supplementary Figure 1.** Immunoblots with antibody for pRXRα, RXRα and GAPDH from (A) insulin- or vehicle-treated brown pre-adipocytes that were non-transfected (none) or transfected with either non-targeting control (NTC) siRNA or *Rxra* siRNA, and from (B) insulin- or vehicle-treated brown pre-adipocytes where the pRXRα antibody was pre-incubated with either no peptide (NP), RXRα phosphopeptide (PP), or RXRα non-phosphopeptide (NPP). Two pRXRα bands (56 and 65 kDa) and total RXRα are quantified, and the presented values are means ± SE; 𝑛 = 3. (C) Quantification of immunoblots with antibody for pRXRα (56 kDa), pAKT, pERK, and pIR is shown for BAT from mice that were fasted or refed for 2 or 6 hours; 𝑛 = 6; one-way ANOVA and Dunnett's multiple comparisons test. (D) Total body weight and fasting blood glucose for CD- or HFD-fed mice; 𝑛 = 6; two-tailed t-test. (E) Quantification of immunoblots with antibody for pRXRα (56 kDa), pAKT, pERK, and pIR is shown for BAT from mice that were fed with CD or HFD and were injected with insulin or saline; 𝑛 = 5-6; two-way ANOVA and Šídák’s multiple comparisons test. All phosphoprotein band intensities are normalized to the total amount of each respective protein, except in the knockdown experiment where pRXRα and RXRα are quantified separately. Asterisk (*) represents significant difference (p < 0.05) from vehicle-treated control cells, fasted mice, saline-injected control mice, or CD-fed mice. Hash (#) represents significant difference (p < 0.05) from cells transfected with NTC siRNA, membrane pre-incubated without peptide (NP), or insulin-injected CD-fed mice.

**Supplementary Figure 2**

**Supplementary Figure 2.** (A) Immunoblots and quantification from WT, *Irs1*^-/-^, and *Irs2*^-/-^ brown pre-adipocytes treated with IGF-1 or vehicle; 𝑛 = 2-3. (B) Quantification of pRXRα, pERK, and pAKT from WT, *Irs1*^-/-^, and *Irs2*^-/-^ brown pre-adipocytes treated with either vehicle or insulin; 𝑛 = 3. (C) Quantification of pRXRα (65 kDa), pIR, pERK, and pAKT from brown pre-adipocytes that were pre-treated with vehicle (DMSO), AKT inhibitor (MK-2206), or MEK inhibitor (U0126), and then stimulated with insulin or vehicle; 𝑛 = 3. (D) Quantification of pRXRα (65 kDa), pIR, and pERK from brown pre-adipocytes at day 0, 2, 4, or 6 of differentiation that were stimulated with insulin or vehicle; 𝑛 = 3. All phosphoprotein band intensities are normalized to the total amount of each respective protein. The presented values are means ± SE. Asterisk (*) represents significant difference (p < 0.05) from vehicle-treated control cells. Hash (#) represents significant difference (p < 0.05) from WT cells, DMSO-treated cells, or D0 cells.

**Supplementary Figure 3**

**Supplementary Figure 3.** (A) Immunoblots using clonal WT brown pre-adipocytes and two independent *Rxra*^-/-^ clones that were stably transfected with EV or *Rxra*WT and treated with insulin or vehicle. Quantification of the bands are shown with pRXRα and RXRα bands separately quantified, and pERK normalized to ERK. The presented values are means ± SE; 𝑛 = 3. (B) Immunoblots using clonal WT brown pre-adipocytes transfected with EV, and *Rxra*^-/-^ brown pre-adipocytes transfected with either EV, *Rxra*WT, or *Rxra*SA, all of which were either insulin- or vehicle-treated. (C) Quantification of pRXRα (65 kDa) immunoblot from WT brown pre-adipocytes transfected with EV, and *Rxra*^-/-^ cells transfected with either EV, *Rxra*WT, or *Rxra*SA, at day 0 or day 6 of differentiation; 𝑛 = 4; two-way ANOVA and Tukey’s multiple comparisons test. Asterisk (*) represents significant difference from vehicle-treated control cells or from sample indicated with the scarab (¤) sign. Hash (#) represents significant difference (p < 0.05) between *Rxra*^-/-^ WT and SA.

**Supplementary Figure 4**

**Supplementary Figure 4.** (A) Venn diagrams showing the number of genes that are closest to being differentially expressed (uncorrected p values < 0.05) in each *Rxra*^-/-^ clone re-expressing *Rxra*WT versus *Rxra*SA. The diagrams show overlapping genes at all time points during differentiation together, or for each day of differentiation separately. (B) Graphs are showing log2 of the transcript counts per million (CPM) for six of the genes that the *Rxra*^-/-^ clones have in common in the Venn diagrams.

**Supplementary Table 1.** Primary antibodies for immunoblotting.

| **Antigen** | **Catalog #** | **Company** |
| --- | --- | --- |
| pRXRα | - | Thermo Fisher Scientific |
| RXRα | sc-553 | Santa Cruz Biotechnology |
| RXRα | #3085 | Cell Signaling Technology |
| pERK | #9101 | Cell Signaling Technology |
| ERK | #4696 | Cell Signaling Technology |
| pAKT | #9271 | Cell Signaling Technology |
| AKT | #9272 | Cell Signaling Technology |
| pIRβ | #3024 | Cell Signaling Technology |
| IRβ | #3025 | Cell Signaling Technology |
| GLUT4 | PA1-1065 | Thermo Fisher Scientific |
| FAS | #3189 | Cell Signaling Technology |
| GAPDH | #2118 | Cell Signaling Technology |
| Vinculin | #13901 | Cell Signaling Technology |

**Supplementary Table 2.** Primer sequences for qPCR.

| **mRNA** | **Forward primer** | **Reverse primer** |
| --- | --- | --- |
| *Rn18s* | AGTCCCTGCCCTTTGTACACA | GATCCGAGGGCCTCACTAAAC |
| *Tbp* | ACCCTTCACCAATGACTCCTATG | ATGATGACTGCAGCAAATCGC |
| *Fabp4* | CTGGGCGTGGAATTCGAT | GCTCTTCACCTTCCTGTCGTCT |
| *Adipoq* | GATGGCACTCCTGGAGAGAA | TCTCCAGGCTCTCCTTTCCT |
| *Slc2a4* | GTGACTGGAACACTGGTCCTA | CCAGCCACGTTGCATTGTAG |
| *Fasn* | GGCTCTATGGATTACCCAAGC | CCAGTGTTCGTTCCTCGGA |
| *Cebpa* | CAAGAACAGCAACGAGTACCG | GTCACTGGTCAACTCCAGCAC |
| *G0s2* | AGGAGATGATGGCGCAGAAG | GTCTCAACTAGGCCGAGCAC |
| *Ndufb3* | ATCCATGGGCTCGCAATGAG | AGCTACCACAAACGCAGCAA |
| *Pparg2* | GCATGGTGCCTTCGCTGA | TGGCATCTCTGTGTCAACCATG |
