# Supplementary figures and images for: Insulin-induced serine 22 phosphorylation of retinoid X receptor alpha is dispensable for adipogenesis in brown adipocytes
Source: Adipocyte. 2020 Apr 5;9(1):142–52. doi: 10.1080/21623945.2020.1747352 (PMC7153655; doi:10.1080/21623945.2020.1747352)

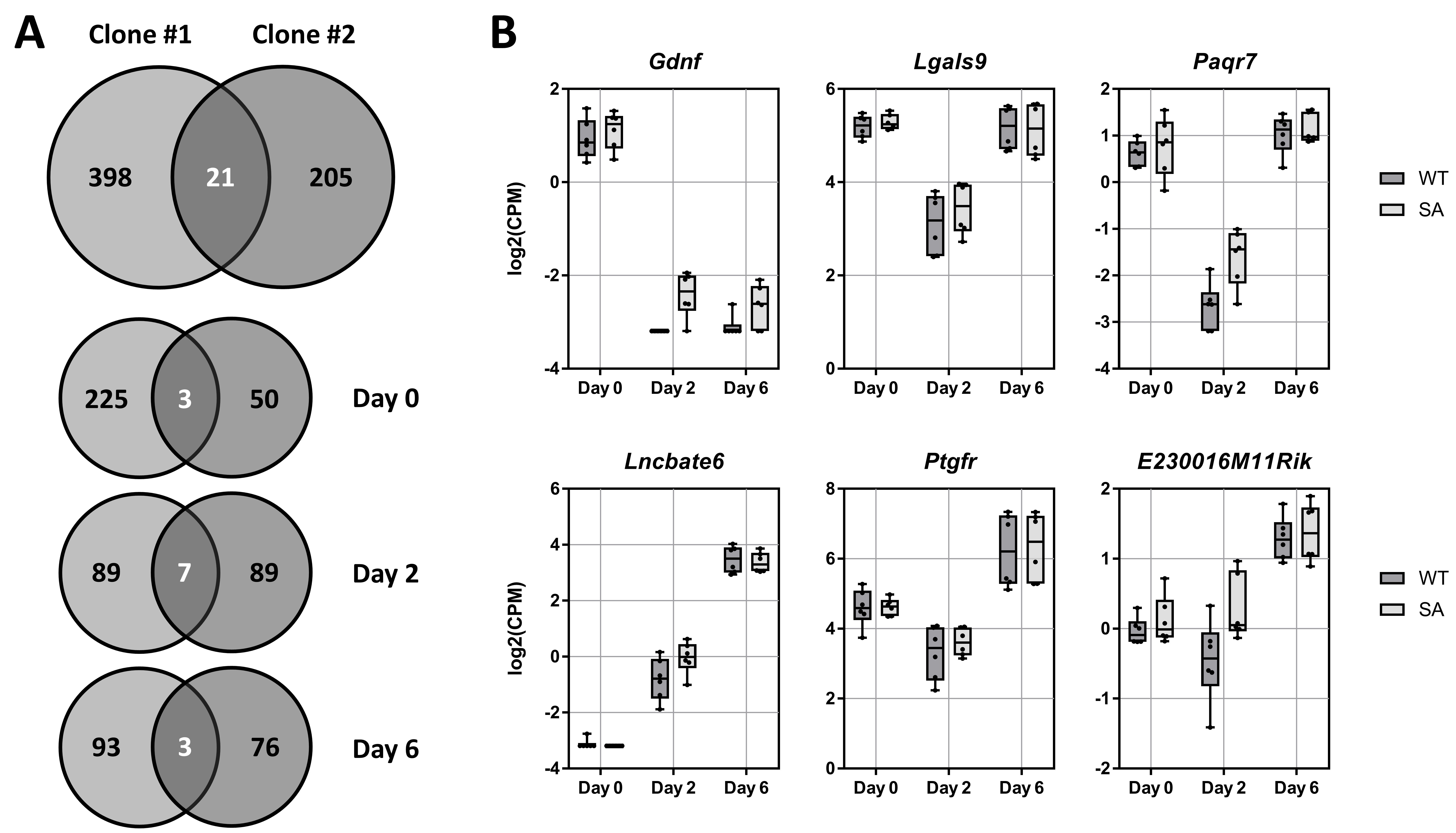

Supplement: Supplemental Material [file KADI_A_1747352_SM0410.zip › Supplementary_Figure_4.jpg]
